# Surviving Deadly Lung Infections: Innate Host Tolerance Mechanisms in the Pulmonary System

**DOI:** 10.3389/fimmu.2018.01421

**Published:** 2018-06-22

**Authors:** Meredith J. Crane, Kayla M. Lee, Ethan S. FitzGerald, Amanda M. Jamieson

**Affiliations:** Division of Biology and Medicine, Department of Molecular Microbiology and Immunology, Brown University, Providence, RI, United States

**Keywords:** host tolerance, pneumonia, lung infections, innate immunity and responses, lung epithelium, lung endothelium, tissue repair and regeneration

## Abstract

Much research on infectious diseases focuses on clearing the pathogen through the use of antimicrobial drugs, the immune response, or a combination of both. Rapid clearance of pathogens allows for a quick return to a healthy state and increased survival. Pathogen-targeted approaches to combating infection have inherent limitations, including their pathogen-specific nature, the potential for antimicrobial resistance, and poor vaccine efficacy, among others. Another way to survive an infection is to tolerate the alterations to homeostasis that occur during a disease state through a process called host tolerance or resilience, which is independent from pathogen burden. Alterations in homeostasis during infection are numerous and include tissue damage, increased inflammation, metabolic changes, temperature changes, and changes in respiration. Given its importance and sensitivity, the lung is a good system for understanding host tolerance to infectious disease. Pneumonia is the leading cause of death for children under five worldwide. One reason for this is because when the pulmonary system is altered dramatically it greatly impacts the overall health and survival of a patient. Targeting host pathways involved in maintenance of pulmonary host tolerance during infection could provide an alternative therapeutic avenue that may be broadly applicable across a variety of pathologies. In this review, we will summarize recent findings on tolerance to host lung infection. We will focus on the involvement of innate immune responses in tolerance and how an initial viral lung infection may alter tolerance mechanisms in leukocytic, epithelial, and endothelial compartments to a subsequent bacterial infection. By understanding tolerance mechanisms in the lung we can better address treatment options for deadly pulmonary infections.

## Introduction

The ultimate goal for a host when responding to an infection is survival and a rapid return to a homeostatic state. This can be accomplished in several non-mutually exclusive ways. One is to quickly and efficiently clear the pathogen, and thus prevent excessive pathogen-induced pathology. The other is to mitigate any damage or changes caused by the infection. The ability to survive an infection is determined by two main factors, pathogen clearance and host tolerance (1–4). Disease tolerance is defined as the ability of the host to tolerate the effects of the pathogens and the potentially damaging effects of the immune response. Problems arise when these strategies are in direct conflict with each other. For example, the immune response in an effort to clear the pathogen often causes damage, that is detrimental to the host. On the other hand, tolerance processes such as anti-inflammatory responses can cause immunosuppression and decrease pathogen clearance. Normally, however, a balance between these two processes is reached, and the infection resolves.

**Figure 1:**
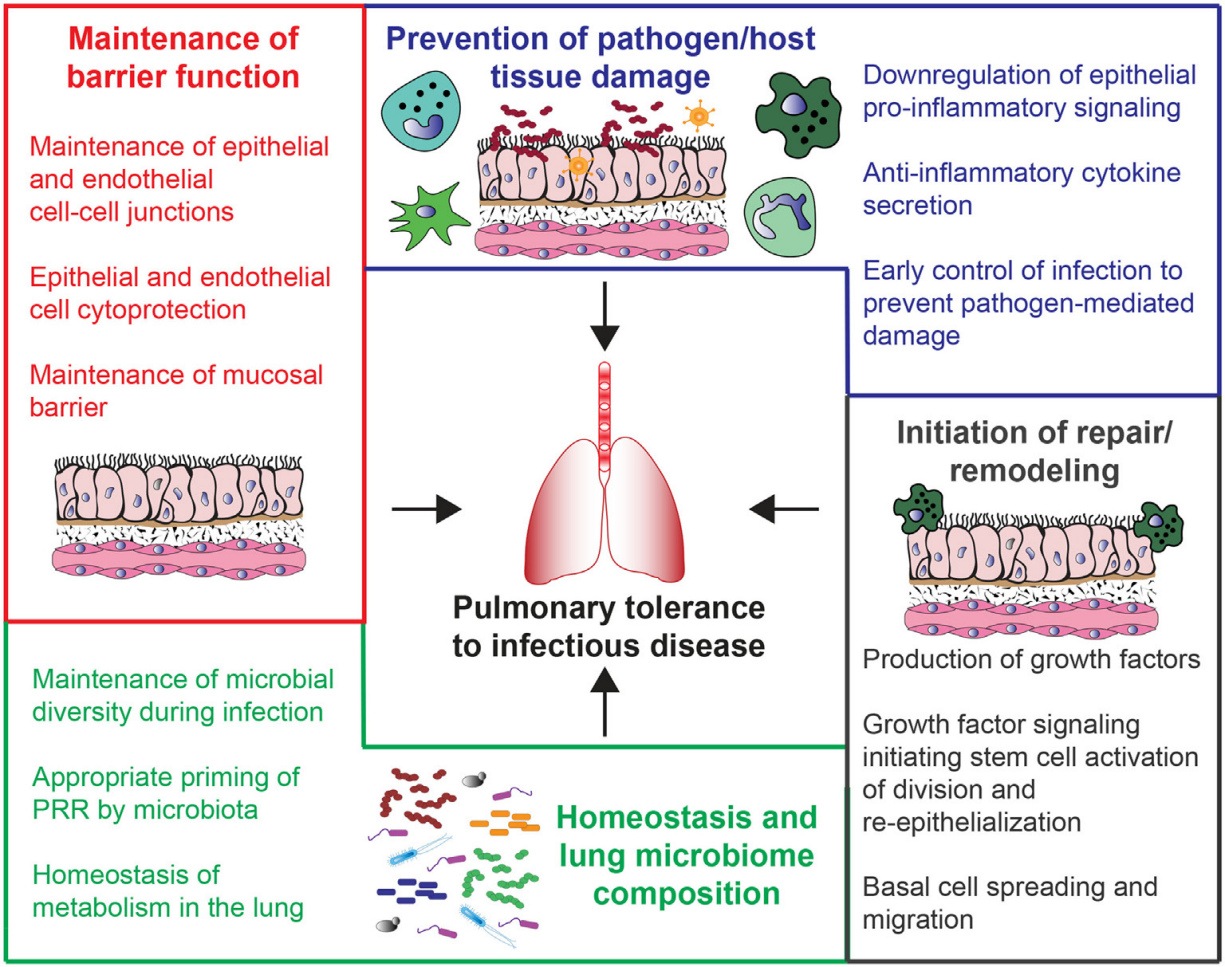
Potential mechanisms of host tolerance to lung infections. These mechanisms are broadly divided into four main categories. Beginning clockwise from the top, they include prevention of pathogen/host tissue damage (blue), initiation of repair/remodeling (gray), changes in lung microbiome composition and homeostasis (green), and maintenance of barrier function (red).

Changes in host disease tolerance are most obvious when the infection is in an essential organ. This is one reason why lung infections are ideal situations to examine mechanisms of tolerance. In addition to increasing our understanding of this aspect of pulmonary disease it also addresses a clinically relevant need (5). Lung infections are a top cause of disease with high economic and humanitarian costs in the United States and worldwide (6, 7). Community-acquired pneumonia (CAP) and hospital-acquired pneumonia (HAP) can be caused by a variety of different pathogens, including viral, bacterial, fungal, and polymicrobial infections (7, 8). Bacterial pneumonia is a common complication of respiratory virus infection that leads to increased morbidity and mortality (9). Given the diversity of pathogens that cause pneumonia, treatment is complex and not always effective (10). As detailed in the recent National Heart Lung Blood Institute Working Group Report, future directions for pneumonia treatment should include host-targeted therapeutics, which includes therapeutics directed at host tolerance mechanisms (11). This review will explore the concept of host disease tolerance mechanisms in the context of acute lung infections (see Figure 1 for a summary).

### Public Health Implications of Lung Infections

Pneumonia is an infection of the lung that causes the alveoli, or air sacs, to fill up with fluid or pus (12). There are several risk factors for the development of pneumonia, such as advanced age, being immunocompromised, or having a pre-existing lung disease. Lower respiratory tract infections (LRTIs) cause the most deaths from an infectious disease worldwide (6), and have a large economic and personal burden (6, 13, 14). Pneumonia is the leading cause of death of children under five years of age worldwide (15). This is particularly true in the developing world, where it causes more deaths than either diarrheal disease or malaria. If pneumonia does not resolve it can lead to acute respiratory distress syndrome (ARDS), sepsis, increased risk of cardiovascular disease, and decreased pulmonary function.

There are several viral infections that lead to pneumonia (16). Influenza A virus (IAV) primarily infects the lung epithelium, and can cause viral pneumonia. It leads to an estimated 500,000 deaths annually, in addition to the hospitalizations and loss of productivity from infected people (17). There are also a variety of other viruses that can infect the lower respiratory tract and lead to pneumonia, including respiratory syncytial virus (RSV), parainfluenza, human metapneumonia, and some adenoviruses (14, 18–22). Rhinoviruses and newly described coronaviruses also infect the respiratory tract and cause disease. RSV, in particular, can cause complications in young children and is the leading cause of hospitalization in children less than one year old in the United States (18). Many of these respiratory viruses spread easily from person to person, or can be spread from an animal reservoir (23).

A variety of bacteria can also lead to the development of pneumonia. Bacterial pathogens as well as opportunistic infections (also known as pathobionts) can lead to pneumonia when allowed to infect the lower respiratory region. Bacteria that cause LRTIs naturally colonize the nasopharynx, but can cause disease when allowed to proliferate in the lower respiratory region (24–28). The most common examples of these are *Streptococcus pneumoniae* and *Staphylococcus aureus*. Other bacteria are acquired from the environment, and often these bacteria have specific virulence factors that allow for the adaptation and infection of the lower respiratory tract (29). An example of an environmental pathogen is *Legionella pneumophila*, which is found in freshwater amoebas and is able to proliferate in alveolar macrophages (30–33). Bacterial pneumonia is a common cause of both CAP and HAP (29, 33, 34). Like viruses, bacteria can also spread from person to person through expelled respiratory droplets.

In addition to viral and bacterial pathogens causing pneumonia there are certain fungal infections that can infect the lower respiratory tract. While more rare than viral or bacterial lung infections, fungal infections of the respiratory tract can be severe and cause pneumonia, especially in immunocompromised patients (35). *Aspergillus, Cryptococcus*, and *Candida* species have all been shown to cause lung infections in certain populations and in certain circumstances, such as individuals with increased environmental exposure and patients with suppressed immune systems (36–38). As there continues to be an increase in immunocompromised populations due to infections, such as HIV and also organ transplant populations, there has been an increase in overall fungal infections (35). Understanding how fungal colonization and infection influence the respiratory tract is an important area of study.

### Polymicrobial Lung Infections

The vast majority of research in infection biology has been devoted to studying the interactions of a single pathogen with a host. In addition to single infections causing pneumonia, a common complication following infection with respiratory viruses is bacterial pneumonia (9, 26, 39–56). Many clinical infections and presumably subclinical infections are often in fact coinfections, in that two (or more) pathogens simultaneously or in close temporal proximity infect a single host (9, 26, 39–59). These infections are termed secondary infection, superinfection, or coinfection. The simultaneous response of two pathogens can manifest in many ways and often results in increased morbidity and mortality. Understanding how an infection with one pathogen can affect the response to another is of paramount importance in the complete understanding of the immune response to infection.

To determine the best treatment options for patients with complex viral/bacterial coinfections increased understanding of the interplay between pathogens and the interaction with the host is necessary. Several viruses and bacteria have been shown to interact to worsen clinical outcomes. It is now believed that most of the deaths associated with the 1918 influenza pandemic were caused by superinfection with bacteria (60, 61). IAV/*S. pneumoniae* coinfection is perhaps the most well-studied example of viral/bacterial coinfection of the lung (62). However, bacterial coinfection also complicates infection with other respiratory viruses, including rhinovirus, metapneumonovirus, RSV, parainfluenza virus, adenovirus, and coronavirus (52, 63–66). Young children are especially vulnerable to bacterial complications following viral infection (44, 62, 67, 68).

There are multiple proposed mechanisms whereby infection with a respiratory virus leads to decreased resistance to bacteria (41–43, 46, 49, 50, 54, 69–73). In most cases examined, initial infection with IAV increases the susceptibility to subsequent bacterial infection (either lung-tropic pathogens or opportunistic commensals), leading to increased bacterial load in the lung and in some cases bacterial dissemination and septicemia. Influenza-induced alterations include a suppression of the pulmonary immune system and changes to the lung epithelium that enable increased bacterial adherence and dissemination. These immunosuppressive mechanisms include neutrophil dysfunction and alterations in expression of essential chemokines and cytokines (41–43, 45, 49, 50, 69–73). Viral neuraminidase alters the lung epithelium causing increased bacterial adhesion (57). While IAV is the best studied and has the clearest causal link to secondary bacterial infections causing pneumonia, several other viruses have also been indicated. RSV has a clear temporal link to causing a secondary pneumonia with *S. pneumoniae* (64, 65). It is likely that most respiratory viruses influence the susceptibility to bacterial infections, either by causing damage or by alteration of the pulmonary immune response.

Bacterial pneumonia secondary to a respiratory virus infection is identified clinically when there is a clear fulminate bacterial overgrowth. This increased pathogen burden correlates with an increased lung pathology, although it is difficult to separate out damage caused by the increased pathogen burden itself from damage caused by the host response. However, there is increasing evidence that alterations in tolerance mechanisms, specifically decreased tissue repair, may play an important role in the pathogenesis of lung infections and this is amplified when the infections are polymicrobial (11, 62, 74–76).

### Host Disease Tolerance to Infectious Disease

Tolerance as a defense strategy against infection was first described by researchers studying infectious diseases in plants. It was based on the recognition that plants could survive an infection by limiting tissue damage, despite having a high pathogen load (77). In subsequent years, tolerance has been recognized as an evolutionarily conserved mechanism for hosts of many species to survive infection and has been described in the context of other infectious diseases (4, 78–80). This includes studies regarding tolerance to infection with plasmodium, the causative agent of malaria (81–83). Unfolded protein responses have been shown to be essential in conferring tolerance to *Pseudomonas aeruginosa* infection, and an increase in tissue repair factors can confer tolerance to lung infections (84, 85). There have also been roles described for the aryl hydrocarbon receptor in controlling the innate immune response that can lead to increased tolerance (86, 87). These and other studies have opened a new line of treatment options for complex infectious diseases.

There are a variety of antimicrobial interventions that have been introduced to combat lung infections. For viruses this includes both preventative vaccines and in some cases antivirals (88–92). There are also a number of antibiotics that target bacterial pathogens and pathobionts that cause infection of the lower respiratory tract. However, despite the increased availability of antibiotics, many bacteria are still associated with pneumonia, including *S. pneumoniae, S. aureus, Klebsiella* spp, *Haemophilus influenzae, Moraxella* spp, and *Legionella* spp (9, 39, 47, 50–52, 56). Often antibiotics are ineffective due to resistance or timing of the intervention. In addition, bacteria that are resistant to all antibiotics are emerging. It is becoming increasingly clear that antimicrobial drugs are not universally effective in treating single infections and especially the more deadly polymicrobial infections of the lung (93, 94). Therefore, novel treatment strategies will be necessary to increase our ability to treat pneumonia. This will be especially relevant as we prepare ourselves for the next IAV pandemic, which will likely include a strong burden of secondary bacterial infection (94–96). It appears that during coinfection the balance between pathogen clearance and host tolerance is disrupted even more than during lung infections with a single pathogen (84, 97). In particular, pathogen-induced damage and damage directly from the immune response may cause a decrease in tissue resilience, making it even more difficult to return to a homeostatic state (24, 98, 99).

This review explores tolerance mechanisms that are affected by infections of the lung, with a specific focus on tolerance mechanisms directed by the innate immune response, the lung epithelium, the lung endothelium, and the lung microbiota. One clear mechanism of decreased tolerance is an excess of inflammation. This can come from innate immune cells as well as from the lung epithelium and endothelium. Decreasing inflammation can increase host tolerance in some cases, but this is complex as the inflammatory response is so closely tied to pathogen resistance (5). Many cells of the innate immune response are also important in tissue repair. These include innate lymphoid cells (ILCs) that produce IL-22 and also the growth factor amphiregulin. Both of these factors primarily act on the lung epithelium to initiate and maintain repair processes. Other important innate immune mediators of repair are alveolar macrophages. Maintaining the barrier functions of the lung epithelium and endothelium is essential for host tolerance in order to regulate the influx of inflammatory mediators during infection. These cells can also themselves become activated during infection and contribute to immunopathogenesis through excessive inflammatory cytokine production. Finally, as is becoming increasingly recognized, not only do the mammalian cells have a role in most aspects of our health, but the microbes that share the body also play important functions. It is likely that host tolerance mechanisms are no exception and microbiota of the lung are able to alter tolerance to pulmonary infections, with loss of homeostasis correlating with microbial dysbiosis. This review will cover these aspects of tolerance to acute lung infections with a specific focus on viral/bacterial coinfections and how tolerance is altered by infection with two distinct pathogens.

## Innate Immune Tolerance Mechanisms

Viral infections of the lung are often characterized by early inflammatory responses from both the lung epithelium and innate immune cells in an attempt to clear the virus, as well as resultant damage done to the tissue by both the virus and the immune response mounted to the virus. Without proper compensatory host mechanisms to return to an anti-inflammatory, homeostatic state, and repair the damage done to the tissue following infection, the host becomes susceptible to secondary bacterial infections which are known to increase morbidity and mortality of the host. It is often challenging to fully separate out how the innate immune response impacts resistance mechanisms from how it affects host disease tolerance. The innate immune response, while necessary to clear the pathogen, can cause damage to the tissue thus decreasing tolerance. However, when the innate immune response is suppressed to prevent immunopathology, this can lead to an increase in pathogen load which in turn can also cause tissue damage. In addition, the acquired immune system plays important roles in both resistance and tolerance to pulmonary infections, but its involvement is beyond the scope of this review, which will focus on innate (or early) tolerance mechanisms. This section of the review will explore the impact of the innate immune system on its contribution to host tolerance to pulmonary infections.

### Decrease of Innate Immunity-Induced Damage Can Increase Host Tolerance

Early control of viral replication is mediated by innate immune cells that respond to signals from infected epithelial cells. Among the early-responding cells are natural killer (NK) cells, of which there is a resident population in the lung. NK cells are essential for viral clearance as has been shown in many infectious models, but they have also been implicated in causing severe lung damage. As part of their antiviral response, NK cells produce a great amount of IFN-γ, which contributes to acute lung injury (ALI) and death (100). Studies have shown that either depletion of NK cells, knockout of IL-15 (a cytokine that controls NK cell proliferation), or neutralization of IFN-γ can decrease morbidity and ameliorate the tissue damage done by NK cells during infection with RSV despite an increase in viral burden, indicating that while NK cells are important for control of viral replication, they are also responsible for increased immunopathology in the lung (101, 102). This is not only true for viral infections, but also for bacterial infections (24–28). One example is that in a model of tularemia, mice lacking NKT cells, cells that share properties of both NK and T cells, survive infection better than mice with NKT cells (103). Invariant NKT cells in conjunction with macrophages have also been shown to cause a chronic inflammatory disease following viral lung infection (104) due to persistent activation of the innate immune response. These studies collectively show that, while essential in responding to lung infections, many innate lymphocyte subsets can cause pathology that decreases tolerance to infection.

Other innate immune cells that are implicated in the pathogenesis of viral infections are inflammatory monocytes and macrophages that infiltrate the lung following infection. Monocytes and monocyte-derived cells, such as macrophages and dendritic cells, are important mediators of the inflammatory response to infection. They are also phagocytes that can help control pathogen burden and remove dead cells and debris that accumulate during infection. However, these cells have also been shown to have roles in contributing to an excessive inflammatory response and resultant damage. In a model of IAV infection, blockage of CCR2, the receptor expressed on monocyte-derived cells that facilitates their entry into the lung, results in decreased inflammatory cell infiltrate, inflammation, tissue damage, and mortality without any effect on viral clearance (105, 106). It has also been shown that failure of these cells to induce programmed cell death during the resolution of infection results in unregulated, prolonged inflammation which in turn decreases tolerance (107–111).

Neutrophils are short-lived polymorphonuclear cells that are potent mediators of the inflammatory response very early during infection that are capable of unleashing powerful antimicrobial defenses at the cost of extreme tissue damage. Although these cells are very important for rapid clearance of pathogens, dampening their inflammatory effects has been shown to be beneficial for improving pulmonary function and survival. In a study of rat coronavirus, depletion of neutrophils results in increased mortality due to delayed viral clearance; however, their absence is also associated with decreased inflammation and breakdown of the epithelium (112). Other studies with IAV infection have shown conflicting protective and pathologic roles for excessive pulmonary neutrophilia. One study has shown that increased neutrophilic recruitment to the lung during IAV infection is associated with increased immunopathology attributed to tissue damage done by neutrophil extracellular traps (113). However, other studies have shown that depletion of neutrophils during early IAV infection not only results in increased viral loads but also increased inflammation and decreased epithelial barrier function (114, 115). Another study showed that depletion of MIP-2/CCL8 results in attenuated neutrophil recruitment into the lung which is associated with decreased pathology without a significant effect on viral burden (116).

Alveolar macrophages are the sentinel cells that patrol the lungs and are often first to encounter pathogenic invaders. These macrophages have very important roles in mediating early defense mechanisms as well as facilitating the return to homeostasis during the resolution of infection. Their roles in fighting against viral infections are as yet controversial and appear to be very virus-specific. For instance, depletion of alveolar macrophages during IAV infection exacerbates inflammation and contributes to decreased epithelial barrier function and vascular leakage (117, 118). Similarly, a model of lung infection with RSV demonstrates increased viral titers, inflammatory cell infiltrate, and resultant inflammation following depletion of alveolar macrophages (119). In contrast, depletion of alveolar macrophages during pulmonary infection with coronavirus is shown to decrease viral titers and increase survival potentially through attenuation of pathogenic T cell responses (120). In addition, depleting alveolar macrophages prior to infection with human metapneumovirus ameliorates disease through significantly decreased viral titers and decreased inflammation (119). Mice with a defect in alveolar macrophages but intact adaptive immunity had normal viral clearance but increased morbidity and lung failure (121). Therefore, the pathogenic or protective contributions of alveolar macrophages appear to depend heavily on the specific viral infection.

### Modulating Tolerance Mechanisms to Infections Can Impact Disease Outcomes

There are several mechanisms that the host employs during the resolution of infection to repair lung injury and it has been shown that the absence or impairment of some of these results in worsened disease outcomes and greater susceptibility to secondary infection. Cytokines and growth factors produced by the innate immune response play a crucial role in suppressing inflammation, initiating tissue repair, and returning the pulmonary system to a state of homeostasis after the resolution of the infection. This section describes the innate immune-produced mediators of tolerance in the pulmonary system.

An important example of this is the role of IL-22 in influenza infection. IL-22 is a cytokine that is expressed by a number of immune cell types and acts on the epithelium to induce proliferation and growth, making it an extremely vital player in mediating repair following infection. In models of influenza infection, IL-22^−/−^ mice exhibit increased morbidity and mortality correlative with decreased airway epithelial integrity and increased apoptosis of epithelial cells during the resolution of infection (122, 123). Importantly, influenza-infected IL-22^−/−^ mice show no difference in viral load when compared to wild-type controls indicating that the decreased survival in these animals is due to decreased tolerance and is independent of resistance to the virus. Conventional NK cells were shown to be a major source of IL-22 during influenza infection and adoptive transfer of IL-22-competent conventional NK cells to IL-22^−/−^ mice was shown to rescue epithelial cell regeneration (124). Another mechanism to promote tissue repair following infection is the activity of amphiregulin, which acts on the epithelium to induce cell proliferation much like IL-22. Studies have identified that during influenza infection, amphiregulin is produced by both ILCs and CD4^+^ regulatory T cells (85, 125). These studies have shown that depletion of either of these cell types or inhibition of their ability to produce amphiregulin results in decreased lung function and epithelial barrier integrity without any changes to viral burden. Administration of amphiregulin in either of these cases was shown to ameliorate tissue damage and facilitate tissue homeostasis (85, 125). Collectively, the research done with both IL-22 and amphiregulin provides examples of the importance of host tolerance in maintaining barrier function in the lung and returning to homeostasis in order to promote survival following infection independent from resistance.

The significance of host tolerance during viral infection is especially highlighted by studies that have shown that in its absence, virally infected hosts become more susceptible to secondary bacterial infections. For example, it has been shown that IL-22^−/−^ mice previously infected with influenza are more susceptible to a secondary bacterial infection with *S. pneumoniae* and exhibit decreased survival and increased bacterial burdens when compared to wild-type coinfected animals (123). In a model of coinfection with IAV and *L. pneumophila*, coinfected mice were shown to have significantly increased morbidity and mortality accompanied by excessive inflammation and tissue damage despite similar viral and bacterial burdens when compared to singly infected animals (84). These effects were abrogated by dampening inflammation through the use of an attenuated bacterial strain combined with administration of amphiregulin, which was able to increase survival and ameliorate damage to the epithelium during coinfection (84). Macrophages also play an important role in regulating tolerance after lung damage (126). Forms of tissue remodeling, such as the remodeling of the extracellular matrix (ECM) during IAV infection, have been shown to critically affect host tolerance (127). Influenza/*S. pneumoniae* coinfections were shown to significantly upregulate MT1-MMP9 expression by macrophages, which contributed to the host-mediated degradation of the ECM and the epithelial cell barrier built upon it. Inhibition of MMP9 by antibody-mediated inactivation was able to significantly limit mortality in mice (127).

Another target for therapy is pattern recognition receptors (PRR). The idea is that by targeting PRR signaling, the damaging aspects of inflammation can be mitigated. Notably, researchers employed the TLR4 inhibitor eritoran in a murine model of lethal influenza infection. Through the first four days of infection and co-treatment with eritoran, viral titers did not notably decrease; however, pro-inflammatory cytokines and chemokines, such as TNF-α, IL-1β, IL-6, and CXCL1 were mitigated during this early infection and the IAV infection resolved more rapidly (128). Similarly, activation of the inflammasome is crucial for clearance of many lung pathogens; however, delaying the activation of NLRP3 during influenza infection not only decreases inflammation but also decreases bacterial burden with a secondary infection (129).

It is important to note that in some cases factors that modulate tolerance have dual roles that can also play a part in altering the outcome of disease. One example of this is shown in a study by Liu *et al*. which demonstrates the interconnectedness of tolerance and resistance. In this study, IL-27 was administered either during the early or late phase of influenza infection and was shown to have a profoundly different effect depending on the time in which it was given. When IL-27 was administered early in infection, it resulted in impaired viral clearance and worsened disease; however, when administered late in infection, there was decreased pathology, increased survival, and no impact on viral clearance. Other examples of factors that modulate both tolerance and resistance in this kind of reciprocal fashion are TGF-β, IL-10, and interferons, particularly type III (72, 130–143).

These results indicate that although some factors may be able to boost tolerance, they also have the potential to negatively impact resistance and are, therefore, perhaps unsuited for therapeutic use in certain infections. On the other hand, there are some factors that play roles in boosting both tolerance and resistance, making them potentially more attractive for therapeutic use. Examples of these can be found in resolvins which have been shown to decrease inflammation in both long-term and acute bacterial infections as well as viral/bacterial coinfections (144–149). In some cases, these lipid mediators can increase resistance to pathogens as well (147, 149, 150).

Taken together, work done in this field has shown that the early immune response to pulmonary infections can damage host tissue, causing loss of tolerance and potentially increasing susceptibility of the host to secondary infections. Inflammation and tissue damage caused throughout a pulmonary infection without proper compensatory tolerance mechanisms in place to ensure the return to homeostasis is associated with decreased survival and increased vulnerability to bacterial infections, and this phenomenon is seen irrespective of control of pathogen burden. In addition, the innate immune response has many factors as discussed above that act to decrease the inflammatory response and/or repair tissue damage. How these factors contribute to tolerance mechanisms in the lung epithelium will be discussed further in the next section. Recent work has emerged that highlights the previously unappreciated role of host tolerance to infections; however, more research needs to be done in order to fully elucidate further mechanisms of immune-mediated host tolerance and the roles that leukocytes play throughout both single and polymicrobial infections.

## Lung Epithelium Tolerance Mechanisms

Epithelial cells represent critical signaling nodes which are responsible for the orchestration of both intracellular and intercellular immune and tolerance responses throughout all stages of infection in the lung (151). Dysregulation of these processes by epithelial cells during single and polymicrobial infections is a major factor in the loss of pulmonary tolerance during infection. This section will briefly describe the broad-ranging responsibilities of epithelial cells signaling in response to general pulmonary infections, from initial sensing to resolution, before highlighting several common mechanisms through which polymicrobial infection dysregulates or abuses these signaling networks to compromise host tolerance (see Table 1 for a summary).

**Table 1:** Summary of epithelial-mediated tolerance responses.

Epithelial-mediated tolerance response	Protein mediators of epithelial cell tolerance response	Pathogens negatively impacting epithelial cell tolerance response
Modulation of pattern recognition and downstream signaling	Toll-like receptors (154), NOD-like receptors (154), RIG-I-like receptor (154)	Respiratory syncytial virus (RSV) (156), influenza A virus (IAV) (156), Sendai virus (156)

Inflammatory upregulation	Type 1 IFN (153), TNF-α (128), IL-1β (128), NF-κB (154), IFN-γ (100)	IAV (128), RSV (100), cytomegalovirus (CMV) (165), Epstein–Barr virus (165), variola virus (165), severe acute respiratory syndrome coronavirus (165)

Barrier function maintenance	Claudin (193), occludin (193), E-cadherin (200), catenin (200)	IAV/*S. pnemoniae* (183), adenovirus (196), coxsackievirus (196), RSV (190, 196), *Haemophilus influenzae* (198), rhinovirus (201), *S. aureus* (198, 202), *P*. *aeruginosa* (190)

Antimicrobial peptide secretion	Glycoconjugated mucins (161, 186), β-defensins (161, 189), surfactant protein D (161)	IAV, *S. pnemoniae* (187), RSV (177, 189), *H. influenzae* (190), rhinovirus (180)

Immune cell recruitment	Type 1 and 2 IFN (147), CCL5, CCL2, CCL8 (117)	IAV (105, 106, 117, 176), IAV/*S. pneumoniae* (148), *F. tularensis* (103), IAV/*L. pneumophilia* (84)

Resolution of clearance effectors	Resolvins (167, 168), TGF-β (136), IFN-λ, IL-22 (122, 123), IL-10	IAV/*S. pneumoniae* (179), RSV (100, 101)

Increased proliferation, differentiation, and repair	TGF-β (136), AREG (85, 125), IL-22 (122, 182), IFN-λ, Fgfr2b (184), ADAMTS4 (186)	IAV/*S. pneumoniae* (43, 123, 178), IAV (124, 179), RSV (180), *P. aeruginosa* (145)

*Potential epithelial-mediated tolerance responses are summarized. The epithelial-derived mediators, and the pathogens that impact these mediators, are described*.

### Epithelial Cells Modulate the Local Pulmonary Immune Response During Acute Infection

A critical first step in any security system, including the immune response, is to detect the presence of intruders. Airway epithelial cells are responsible for the detection of microbes in the respiratory system *via* the recognition of pathogen-associated molecular patterns (PAMPs) and damage-associated molecular patterns (DAMPs) (152). Epithelial cells accomplish this through the expression of a diverse repertoire of PRRs, such as toll-like receptors (TLR), C-type lectin receptors, cytoplasmic retinoic acid-inducible gene-I-like receptors, and NOD-like receptors (153). However, modifications in the expression levels of PRRs in response to primary infections can lead to profound diminishments in tolerance for secondary infections. A wide range of viruses upregulate type I IFN expression in respiratory epithelial cells, which correlates with a significant upregulation of TLRs in many lung resident cells, including respiratory epithelium (154, 155). This dramatic upregulation of TLRs in response to the viral infection has been hypothesized to contribute to the upregulation of cytokine secretion and the initiation of cytokine storm and ARDS. Many therapeutic strategies inhibiting either the activity or signaling downstream of PRRs have been shown to augment host tolerance to secondary bacterial infection through such mechanisms (128, 156).

Upon detection of PAMPs by PRRs, respiratory epithelial cells trigger a broad battery of inflammatory genes and type 1 IFN downstream of NF-κB and the IRF transcription factors, respectively, which has been reviewed extensively elsewhere (152, 157–159). Generally, PRR signaling upregulates cell-autonomous and non-cell-autonomous immune responses to infection. Cell-autonomous functions include the secretion of antimicrobial peptides (AMPs) by epithelial cells, programmed cell death, and other intracellular stress response pathways (145, 160, 161). Non-cell-autonomous signaling primarily works through the initial secretion of cytokines mediating immune cell recruitment (162). However, as has been well-documented in the case of influenza/bacterial coinfection, priming of the immune response by initial influenza infection results in a massive over-recruitment of immune cells by epithelial cells. This occurs due to the cytokine storm generated by epithelial cells, which are primed and actively secreting cytokines to respond to the primary infection, and become hyper-stimulated upon sensing of PAMPs and DAMPs generated by the secondary bacterial infection (163). Similar responses occur with pulmonary infections by cytomegalovirus, Epstein–Barr virus, *Streptococcus spp*, variola virus, severe acute respiratory syndrome coronavirus, and many others (164). Oftentimes, such complications will present as ARDS in the clinic due to diminished pulmonary tolerance when responding to simultaneous infections (164).

Once immune cells are in the pulmonary environment, respiratory epithelial cells further modulate their behavior by signaling through more cytokines, alarmins, and efferocytic signals to augment clearance efforts (165–167). Finally, upon clearing the infection, respiratory epithelial cells direct the resolution of the immune response through the secretion of resolvins to enable cells in the pulmonary space to transition their efforts from clearance to repair and remodeling to restore pulmonary homeostasis (146, 166–169).

Oftentimes, viral respiratory pathogens will take advantage of the proliferative state that epithelial cells enter during remodeling and repair efforts to augment their own proliferation in the cell. For instance, IAV has been observed to induce epithelial cell expression of TGF-β and processing of latent TGF-β precursor into active TGF-β to both suppress the host immune response and to enhance its own replication (136, 170). Add-back therapeutic strategies introducing exogenous resolvins into the respiratory space have also been observed to augment tolerance in certain instances when they do not compromise pathogen clearance (144, 146).

### Alteration of the Lung Epithelium During Infection

Respiratory epithelium tissue homeostasis is required to maintain the continuous biomechanical and cellular processes associated with aerobic respiration. However, the respiratory epithelium is also one of the primary tissue types affected by pulmonary infection, with dysfunction and degradation of the epithelial layer being a primary mechanism of pathogenesis. The reason for this is that lung epithelial cells are the primary target for infection in diverse respiratory viral diseases, such as IAV (171), RSV (172), coronavirus (173), rhinovirus (174), parainfluenza virus (175), and respiratory adenovirus (176).

As a target for lung pathogens, the respiratory epithelium plays an important role in pathogen-sensing and orchestrating downstream inflammatory responses (151). The multifaceted immune response of the respiratory epithelium must strike an appropriate balance between pro-inflammatory mechanisms of pathogen clearance that may cause incidental tissue damage and anti-inflammatory mechanisms of cytoprotection and tissue regeneration which can inhibit clearance. While this is true for certain viral, bacterial, and fungal pathogens, it is amplified in coinfection. Current research into respiratory viral/bacterial coinfections indicates that much of the enhanced pathogenicity of these coinfections stems from the inability of respiratory epithelial cells to triage these immune responses to simultaneous respiratory infections while incurring severe damage (177). Fortunately, the pulmonary research community has made significant strides in understanding the immune mechanisms underlying tolerance to respiratory infections by altering components of the respiratory epithelium’s response to infection regarding pro-inflammatory and cytoprotective signaling. The following section will review recently identified mechanisms of respiratory epithelial tolerance and their potential significance as therapeutics mitigating the severity of diverse pulmonary infections.

### Tissue Repair/Cytoprotection-Mediated Tolerance

Host tolerance is the ability of the host to sustain an ongoing infectious state characterized by high pathogen titers, while maintaining tissue integrity and homeostasis. This allows for proper organ function and the avoidance of pathogen-mediated symptomology, morbidity, and mortality. With most respiratory infections, much initial pathology results from respiratory epithelial cell death. IAV/bacterial coinfections are an excellent case study in this phenomenon. IAV and a range of bacterial coinfections exhibit synergistic lethality resulting from a combination of IAV’s initial infection compromising the respiratory epithelium of the host and the subsequent inability to initiate tissue repair due to uncontrolled inflammation and tissue damage incurred while simultaneously combating the secondary bacterial infection (178). IAV initially infects the upper respiratory tract and spreads to the lower respiratory tract within the first several days of infection (178). Infected cells throughout the respiratory epithelium become dysfunctional due to the burdens of intracellular viral replication, resulting in denuding of the respiratory epithelium and exposure of the basement membrane (165). This primes the respiratory environment for the emergence of opportunistic bacterial infections (pathobionts), or infection by bacterial pathogens. Denuding the epithelial layer exposes matrix proteins which contain an array of receptors for bacterial adherence, such as the adherence of *S. pneumoniae* to the tracheal epithelium of IAV-infected mice (166). Bacterial infection of the newly exposed basal layer prevents the initiation of epithelial coordinated tissue repair (178). To clear the bacterial infection, the epithelium recruits immune cells. This response can trigger a severe inflammatory response, further damaging the pulmonary tissue, while worsening the overall progression of the disease state and inhibiting the initiation of the repair process by epithelial cells. In sum, there is a severe loss of host tolerance to IAV/bacterial coinfection resulting from the initial cell death caused by IAV. This allows for the secondary infection and the subsequent over-recruitment of immune cells, which secrete pro-inflammatory cytokines that interfere with the initiation of tissue regeneration and repair.

Augmentation of respiratory epithelial cell cytoprotection and tissue repair has become a central theme in the search for host-directed therapeutic strategies increasing tolerance to pulmonary infection. The role of ILCs targeting tissue repair was described above (85, 125). While pathways inducing cytoprotection or inhibiting cell death are often separate from pathways involved in tissue repair, tolerance is often maximally impacted by inducing both effects simultaneously. Previous research has demonstrated that modulating inflammatory responses by blocking TLR signaling and upregulating tissue repair through amphiregulin treatment significantly increases host survival in a model of IAV and *L. pneumophila* coinfection (84). Inhibition of PRRs and their downstream signaling is capable of significantly suppressing the inflammatory response. However, PRR activation is also a critical trigger initiating bacterial clearance by epithelial and immune cells. Inhibiting the inflammatory response to augment host tolerance is a delicate balance as described in the innate immune section of this review.

It is a general consensus that many respiratory pathogens have evolved strategies to prevent host tissue repair and the return to epithelial homeostasis. This allows them to maintain an environment conducive to pathogen replication (179–181). Many of these tissue repair strategies are started by the innate immune response (as described above), but their effects are upon the lung epithelial cells. Transcriptomics analysis of the 2009 pandemic IAV infection with *S. pneumoniae* coinfection demonstrated that the two pathogens interacted synergistically to significantly downregulate tissue remodeling, epithelial cell proliferation, and cytoprotective transcriptional pathways (43). Many studies have also demonstrated that restoration of critical signal transducers in these repair pathways, such as IL-6 (182), IL-22 (122), Fgf10 (183), and ADAMTS4 (184), are able to restore repair and help to rescue murine models of IAV infection *via* augmented host tolerance. In particular, Barthelemy *et al*. demonstrated that the increase in tissue integrity resulting from IL-22 immunotherapy reduces secondary bacterial systemic invasion (185). Small molecule therapeutics, such as progesterone, which acts on the amphiregulin pathway to initiate tissue repair after IAV infection, have also been investigated with some success in a female murine model (186). Characterizing discrete host tolerance pathways modulating tissue repair and cytoprotection is required to effectively develop tolerance-augmenting therapeutic agents.

### Modulation of Respiratory Epithelial Barrier Dynamics

The maintenance of barrier function between the lumen of the lung and the bloodstream is one of the primary functions of respiratory epithelial cells and is critical in tolerance of pulmonary infection. The direct infection of the respiratory epithelium by lung pathogens and commensals is normally prevented by the presence of the mucosal layer containing secreted AMPs, such as β-defensin, MUC5AC, and MUC5B (187). While it has long been clear that the mucosal layer is critical in regulating tolerance and tissue homeostasis, recent findings have further elucidated mechanisms through which it modulates host tolerance to infection. For instance, mucins and many other AMPs are interspersed with other glycoconjugates in the respiratory mucosa (188). Mucins themselves are also highly sialylated, assisting in the formation of the mucosal barrier (188). However, influenza viral neuraminidase is capable of penetrating through the respiratory mucosal layer and infecting mucus-producing epithelial cells by cleaving sialylated glycoconjugates (189). This results in an inability to maintain the density of the mucosal layer, which promotes the transition of bacteria that are found to colonize healthy individuals to overgrow and become pathogenic (26, 190). It was demonstrated that the impact of influenza infection was modulated by the concentration of sialic acid content in mucins, and increasing the concentration of sialylated substrates in mucins increased the resistance of epithelial cells to influenza infection in a dose-dependent manner (189). Primary RSV infection has also been shown to downregulate the transcriptional expression of β-defensin, which allows for *H. influenzae* to transition from commensal to pathogenic in the upper airway, by inhibiting the microbicidal activity of the mucosal layer (191).

Not only do direct interactions between pathogens and mucosa mitigate the tolerance effects of the mucosal barrier, but indirect effects of primary viral infection on the composition of the respiratory epithelial barrier and behavior of respiratory immune cells also negatively impact mucosal-mediated tolerance. RSV infection has been demonstrated to infect basal epithelial stem cells which control the ratios of ciliated and mucosal cells in the progeny. RSV-infected basal cells produce many more mucosal cells and far fewer ciliated epithelial cells (177). Therapeutic strategies that speed the regeneration of mucus-secreting epithelial cells could serve to augment tolerance in some infections, but also may worsen outcomes in others. This dysregulation of mucociliary function promotes environments more amenable to secondary bacterial infection. Similarly, rhinovirus has been demonstrated to induce neutrophil elastase, which cleaves and inactivates AMPs secreted into the mucosa by respiratory epithelial cells and promotes secondary bacterial infections, thereby inhibiting the steady state tolerance mechanisms in the respiratory epithelium (192). It was proposed that therapeutics downregulating or inactivating neutrophil elastase during rhinovirus infection might help to maintain host tolerance during the infection (192).

Below the respiratory mucosa, the respiratory epithelial cells themselves also operate as a critical barrier preventing pathogenic infections from spreading beyond the respiratory system into a systemic bacteremia. Maintenance of the respiratory epithelial barrier function below the mucosal layer requires the maintenance of a complex network of intercellular junctions linking individual epithelial cell cytoskeletons. The maintenance of this barrier during infection is a critical tolerance mechanism preventing the dire outcomes resulting from respiratory infections transitioning to systemic bacteremia, pulmonary edema, and excess infiltration of immune cells. Epithelial cell–cell junctions bind epithelial cells into the cohesive barrier between the lumen of the epithelium and the parenchyma. Respiratory epithelial junctions have been extensively reviewed elsewhere (193–195). This section serves to summarize their function and relevance in the maintenance of epithelial barrier function and host tolerance to pulmonary infection.

Tight junctions form the separation between the apical and basolateral face of epithelial cells. Integral membrane components of tight junctions are mainly comprised of claudins, occludins, and adhesion molecules (194, 196). However, tight junctions are highly heterotypic with many different constituent components. Tight junctions often contain many entry receptors for pathogens. When components of tight junctions are employed as entry receptors, their ability to effectively maintain barrier function decreases and diminishes the host’s ability to tolerate secondary bacterial infections as effectively. This dynamic has been observed in both models of adenovirus and coxsackie virus infection (197). Pathogen modification of gene expression also has the capacity to interfere with tight junction expression. RSV infection was shown to downregulate the expression of *claudin-1* and *occludin* in a mouse model, inhibiting barrier function as mediated by tight junctions (198). Infection with *H. influenzae* was also demonstrated to downregulate host transcription of *e-cadherin* through inhibition of FGF2, mTOR, and Slug (199). IAV infection was also shown to damage respiratory epithelial cell barrier integrity by downregulating the expression of tight junction protein claudin-4 (200). Further research also attributed the loss of tight junction integrity during IAV infection to critical tight junction-associated PDZ proteins (197). Loss of epithelial barrier integrity is a critical component in the migration of bacteria to the bloodstream where they can cause sepsis (188). Influenza-mediated disruption of such tight junctions has been demonstrated to contribute significantly to the onset of ARDS from IAV infection (200).

Adherens junctions are also common targets of microorganisms infecting the lung. Adherens junctions are comprised of E-cadherin and catenin proteins, and serve as critical junctions anchoring epithelial actin cytoskeletons together into a network which generates tensile strength and barrier function, while maintaining the tissue pliability required for the biomechanics of respiration (201). Rhinovirus infection, which is characterized by vascular permeability and associated with bacterial secondary infection (202), has been shown to modify respiratory epithelial cells during infection to lower the transcriptional output of *zo-1, occludin, claudin*, and *e-cadherin* by over 50% individually (202). Another study validated such findings and demonstrated a significant loss in transepithelial resistance during rhinovirus infection that was not mediated by cell death or apoptosis, but an increase in severity of coinfection (203).

Compounds causing an upregulation in gene expression or assembly of junction proteins on respiratory epithelial cells could be promising tolerance-augmenting therapeutics for use during diverse viral primary infections. Many bacterial and fungal pathogens of the lung, including *S. pneumoniae, S. aureus, Candida albicans*, and *P. aeruginosa*, employ adhesion junction components, specifically E-cadherin, as an adherence or entry receptor for invasion and colonization (199). The severity of phenotypes observed due to the alpha-toxin protein of *S. aureus* has also been shown to be modulated by the abundance of functional adherens junctions (204). All of these mechanisms dramatically decrease the host’s ability to tolerate low level infections by escalating the degree of damage caused by these pathogens with a poorly maintained epithelial barrier.

The maintenance of epithelial barrier function is also reliant on the maintenance of epithelial cell viability. Respiratory epithelial cell death can substantially decrease host tolerance by forming gaps in the mucosal barrier, which enables respiratory pathogens to directly infect the basal layer of epithelial cells (178). Once penetrating to the basal layer of respiratory cells, pathogenic bacteria such as *S. pneumoniae* and *P. aeruginosa* can translocate through the basal membrane to initiate bacteremia that can lead to sepsis (205, 206). However, appropriate modulation of respiratory epithelial cell death can also promote pulmonary tolerance. Epithelial cell induction of apoptosis is canonically regarded as a means through which the host can restrict pathogen replication in infected cells, without loss of membrane integrity and the secretion of DAMPs leading to hyper-inflammatory responses by the immune system (207). Many respiratory pathogens compromise pulmonary tolerance through the inhibition of apoptotic cell death and the upregulation of more inflammatory forms of cell death, such as necrosis, oncosis, or pyroptosis (208–211). The hyper-inflammatory response to such forms of cell death is affected by immune cells sensing DAMPs. A balance between the maintenance of cell viability/barrier function and the need to restrict intracellular pathogens’ ability to replicate is required to maximize host pulmonary tolerance.

## Pulmonary Endothelial Cell Tolerance Mechanisms

### Endothelial Barrier Function

The pulmonary endothelium is an important interface between the circulation and the lung tissue and airways. In the homeostatic state, the thin pulmonary endothelium forms a barrier between proteinaceous fluids and leukocytes in the circulation and the lung epithelial layer, which is separated by less than 1 µm in the alveoli. In response to inflammatory stimuli from the lung, as in the event of an infection, this homeostatic state is disrupted when circulating leukocytes are induced to marginate along the vascular endothelium through interactions mediated by adhesion molecules, including selectins and integrins. From there they extravasate into the interstitial space in a process that depends on the loosening of endothelial cell junctions; this modulation of the endothelial barrier function can subsequently tune the magnitude of leukocyte infiltration and, therefore, inflammation in the lung. At the same time, this disruption in the endothelial barrier allows for the movement of protein-rich fluids from the circulation into the lungs, causing edema and, in severe cases, ARDS or ALI (212–215). After infection, a certain degree of vascular permeability is required to facilitate the influx of leukocytes into the lung to allow the inflammatory response to control pathogen elimination; however, if the inflammatory response is too robust, the lung tissue can become severely damaged, as discussed in previous sections. Barrier function is the primary contribution of the endothelial layer to maintaining host tolerance and tissue integrity during pulmonary microbial infection.

Much of what is known about pulmonary endothelial barrier function, and the mechanisms that drive the loss of this function, come from studies of single microbial lung infection. The importance of a functional endothelial barrier was demonstrated in a model of *E. coli* pneumonia, in which blockade of the interaction between integrin αvβ3 and its binding partner IQGAP1 at the endothelial cell–cell junction led to excesses in lung extravascular plasma and water, as well as increased lung weight within just 5 h of infection (216). It has also been shown that influenza infection can lead to vascular leak (217, 218). This loss of barrier integrity stems, at least in part, from active infection of endothelial cells by influenza virus. This has been demonstrated in multiple species, including human, in which the pulmonary microvascular endothelium is permissive to infection with multiple clinical and laboratory strains of influenza (217). Similarly, using a human H1N1 influenza model in ferrets, virus was detected in multiple lung compartments including the vasculature (219). Virus-mediated apoptotic cell death is one way in which infection contributes to loss of endothelial barrier function. Influenza-induced endothelial apoptosis could be ameliorated by inhibition of caspases, thereby restoring barrier function (217). Endothelial apoptosis may be due in part to the induction of TNFR1 receptor expression on the endothelial cell surface by IAV (220). This apoptotic signal was enhanced by the interaction of *S. aureus* protein A and TNFR1 in the event of secondary bacterial infection, leading to eventual development of ARDS (220). This finding illuminates the potential for slight alterations in endothelial cell signaling that are induced during a single infection, such as the induction of caspases or TNFR1, to dramatically reduce the host’s ability to maintain homeostasis in the event of a secondary infection.

Apoptosis-independent effects of infection on loss of endothelial barrier function have also been elucidated. Studies simulating viral infection by stimulating human microvascular endothelial cells with poly(I:C) shed light onto the mechanistic link between viral infection and loss of barrier function by showing that signaling through TLR3 and NF-κB induced a loss of claudin-5 expression, a key protein in the formation of endothelial tight junctions (154). A similar effect was demonstrated by infecting human microvascular endothelium with a replication-deficient influenza virus (217). In this study, UV-inactivated virus was still able to induce loss of endothelial barrier function without causing cellular apoptosis. This was driven by the degradation of claudin-5. Interestingly, treatment with the cAMP analog formoterol could restore claudin-5 protein levels and improve endothelial cell barrier function *in vitro* (217). Formoterol’s barrier-enhancing effect when administered after influenza infection was corroborated *in vivo* (217), raising the interesting possibility that barrier-enhancing drugs may present a viable therapeutic option to boost tolerance to the tissue-damaging effects of lung infection if they can be shown not to alter host resistance to the pathogen.

Drug repositioning to treat infection-induced ARDS due to loss of endothelial barrier function is an enticing clinical option. This has been probed experimentally using the cancer drug imatinib. This tyrosine kinase inhibitor was originally developed to target the BCR-Abl fusion protein causing the development of chronic myeloid leukemia cells. Imatinib also targets other diverse kinases, such as the platelet-derived growth factor receptor, which suggests that the drug also functions in modulating barrier function. A report by Rizzo *et al*. tested imatinib’s function in a model of ALI induced by the combination of LPS and ventilator-induced lung injury. This work found that administration of imatinib reduced multiple measures of vascular permeability including cellular infiltration and total protein and pro-inflammatory cytokine concentrations in the bronchoalveolar lavage fluid. Imatinib was found to act in this model by reducing NF-κB activity, and reduced the symptoms of ALI, even when administered prophylactically (221). A similar effect of imatinib on edema and neutrophil influx was observed in a rat model of ischemia/reperfusion injury (222). Whether this type of treatment has the potential to be beneficial to the maintenance of host tolerance without compromising resistance to infection will need to be explored.

Virus-infected lungs are also prone to thrombus formation along the endothelium, which has downstream effects on vascular permeability. Autopsy examination of lung histology of IAV-infected patients has shown evidence of microthrombi formation along the endothelium (217). Similarly, thrombi were observed in specimens from the 1918 pandemic influenza outbreak, although they were absent in autopsy examinations from the 2009 pandemic (223), suggesting that there are strain-specific effects on this process. It is possible that this is one mechanism of the pathology caused by highly pathogenic avian influenza strains (224, 225). Experimental evidence has shown that platelets adhere to endothelial cells through interactions between platelet integrin α5β1 and endothelial fibronectin during influenza infection (217). This interaction negatively impacted host tolerance during infection, as platelet inhibition was shown to improve survival (217). Thrombus formation has also been observed in coinfection, with activation of clotting factors, coagulation and tissue factor, as well as neutrophil elastase deposition on endothelial cells; together these events could enhance vascular permeability leading to more severe inflammation in coinfected lungs (223).

### Contribution of Endothelial Cells to Cytokine Storm

As has been observed with innate immune cells and the lung epithelium, the endothelium itself can also contribute to loss of host tolerance to infection through excessive induction of cytokines leading to cytokine storm. Influenza infection has been shown in several models to induce upregulation of PRRs and inflammatory cytokine and chemokine production, thereby elevating the risk of inflammation-induced tissue damage. Primary human lung endothelial cells were shown to upregulate transcripts for TLR2 and NOD2 (220). Similarly, infection of ferrets with human H1N1 induced TLR3 expression on endothelial cells (219). Activation of TLR3 on primary human lung microvascular endothelial cells with the synthetic ligand poly(I:C) induced the expression of pro-inflammatory cytokines, including IL-6, IL-8, TNF-α, and IFN-β, leading to the possibility that excessive stimulation could lead to cytokine storm (226). In addition to modulating PRR expression and signaling, influenza infection has been shown to drive cytokine storm through enhanced S1P1 signaling in endothelial cells (226). Cytokine storm following pulmonary infection is detrimental to the host, as limiting its magnitude has been shown to improve survival. For example, limiting H5N1 influenza infection in endothelial cells using microRNAs both reduced cytokine storm and improved survival (218). Similarly, treatment with an S1P1 antagonist during influenza infection reduced mortality in an endothelial cell-specific manner (226). It is reasonable to speculate that induction of inflammatory responses in endothelial cells by a primary viral infection would prime a quicker and potentially more vigorous response to secondary bacterial infection, leading to prolonged cytokine storm with leukocyte infiltration, and ultimately tissue damage and organ failure, and that intervening with these processes could promote tissue protection.

## Lung Microbiome in Host Tolerance

The lung is host to numerous microbiota, and their roles in human health and disease are beginning to be documented (227). Many studies have examined the link between the gut microbiota and pulmonary health; however, that topic is beyond the scope of this review (228, 229). How the commensal microbiota of the lung contribute to host tolerance to infection is not well understood; however, studies are beginning to probe the changes that occur in the lung microbial milieu during active infection, which may shed some light on their roles in tissue homeostasis (28, 230–232). As the previous sections of this review have discussed, the line between what is a commensal bacteria and what is a pathogen in the lung is rather amorphous. This is especially true after preceding viral infections where previously harmless bacteria become pathogenic (233, 234). These opportunistic infections, or pathobionts, comprise the vast majority of secondary bacterial infections following respiratory viral infections.

Regarding the lung microbiome, in a correlative study examining the serial colonization of the nasopharynx of infants in their first year of life, it was found that the onset of viral acute respiratory infections was associated with the transient appearance of *Streptococcus, Moraxella*, or *Haemophilus* species. This study also found that the composition of the microbiome was a determinant of whether disease would spread to the lower airways and cause elevated inflammation or asthma (235). The composition of the airway microbiome is not restricted to only bacterial species. In fact, examination of patients admitted to the ICU revealed an overabundance of *Candida* species that was not dependent on the type of pneumonia or whether the patient had been treated with antibiotics (236). Changes in the composition of lung microbiota have also been monitored in patients with viral and bacterial coinfection. In a study that examined bronchoalveolar lavage fluid samples serially drawn from a H7N9 influenza-infected patient, it was found that the microbiota became dominated by *Acinetobacter baumannii*, which eventually became multi-drug resistant and led to secondary bacterial infection (237). This transition was accompanied by increased inflammation, raising the possibility that there could be an associated increase in lung immunopathology (237). Similarly, comparison of the oropharyngeal microbiome revealed distinct differences in composition between healthy patients and those with H7N9 influenza or H7N9 influenza infection with secondary bacterial infection. In particular, the healthy patients had an enrichment of *Haemophilus* and *Bacteroides* species (238). In contrast, influenza-infected individuals had outgrowths of *Filifactor, Megasphaera*, and *Leptotrichia* species, while the addition of secondary bacterial infection led to further dysbiosis, including the enrichment of *Leptotrichia, Oribacterium, Streptococcus, Atopobium, Eubacterium, Solobacterium*, and *Rothia* species (238).

Documenting the changes in airway microbiota that occur in response to pulmonary infections raises the possibility of using commensal microbes to improve host defense against pathogens. This has been investigated experimentally in a few instances. For example, it has been shown that intranasal administration of *Lactobacillus rhamnosus* can aid in resisting RSV infection in infant mice (239). Another study suggested that this was due to a priming effect, showing that nonviable *L. rhamnosus* or the bacterial cell wall component peptidoglycan can enhance inflammatory responses in a TLR3-dependent manner (240). In a similar manner, TLR3-dependent priming with the upper respiratory tract-resident species *Corynebacterium pseudodiphtheriticum* also improved the outcome of RSV and secondary *S. pneumoniae* infection (241). While these studies emphasized the effect of priming on pulmonary resistance to subsequent infection, it is plausible that these microbiota also influence host tolerance mechanisms. Whether the prevention of commensal dysbiosis during infection can promote pulmonary homeostasis in the face of infectious insult will be an important area for future study.

## Conclusion

The role that host disease tolerance mechanisms play in the ability to survive a lung infection is an important new area of research. This review focused on the interacting roles that the innate immune response, the lung epithelium, and the lung endothelium play when responding to acute lung infections (as summarized in Figure 1). It also demonstrated the complexities that arise in host tolerance to polymicrobial infections, and posed several questions regarding the role of the lung microbiota in tissue protection during infection. An increased understanding of host tolerance to acute lung infections will allow us to not only improve treatments for these deadly diseases but may also open up new treatment options for chronic lung diseases and infections.

## Author Contributions

AJ, MC, EF, and KL researched and wrote the article.

## Conflict of Interest Statement

The authors declare that the research was conducted in the absence of any commercial or financial relationships that could be construed as a potential conflict of interest.
